# Absolute quantification of neuromelanin in formalin-fixed human brains using absorbance spectrophotometry

**DOI:** 10.1371/journal.pone.0288327

**Published:** 2023-07-10

**Authors:** Dipshay Avi Chand, Miriam Scadeng, Birger Victor Dieriks

**Affiliations:** 1 Department of Anatomy and Medical Imaging, University of Auckland, Auckland, New Zealand; 2 Centre for Brain Research, University of Auckland, Auckland, New Zealand; 3 Mātai Medical Research Institute, Gisborne, New Zealand; Federal University of Ceara: Universidade Federal do Ceara, BRAZIL

## Abstract

Parkinson’s disease is characterised by a visual, preferential degeneration of the pigmented neurons in the substantia nigra. These neurons are pigmented by neuromelanin which decreases in Parkinson’s disease. Not much is known about NM as it is difficult to study and quantify, primarily due to its insolubility in most solvents except alkali. Neuromelanin quantification could progress the development of biomarkers for prodromal Parkinson’s disease and provide insights into the presently unclear role of neuromelanin in Parkinson’s disease aetiology. Light microscopy with stereology can visualise pigmented neurons, but it cannot quantify neuromelanin concentrations. Absolute neuromelanin quantification using absorbance spectrophotometry is reported in the literature, but the methodology is dated and only works with fresh-frozen tissue. We have developed a quantification protocol to overcome these issues. The protocol involves breakdown of fixed tissue, dissolving the tissue neuromelanin in sodium hydroxide, and measuring the solution’s 350 nm absorbance. Up to 100 brain samples can be analysed in parallel, using as little as 2 mg of tissue per sample. We used synthetic neuromelanin to construct the calibration curve rather than substantia nigra neuromelanin. Our protocol enzymatically synthesises neuromelanin from dopamine and L-cysteine followed by high-heat ageing. This protocol enables successful lysis of the fixed substantia nigra tissue and quantification in three brains, with neuromelanin concentrations ranging from 0.23–0.55 μg/mg tissue. Quantification was highly reproducible with an interassay coefficient of variation of 6.75% (n = 5). The absorbance spectra and elemental composition of the aged synthetic neuromelanin and substantia nigra neuromelanin show excellent overlap. Our protocol can robustly and reliably measure the absolute concentration of neuromelanin in formalin-fixed substantia nigra tissue. This will enable us to study how different factors affect neuromelanin and provide the basis for further development of Parkinson’s disease biomarkers and further research into neuromelanin’s role in the brain.

## Introduction

Parkinson’s disease (PD) is a neurodegenerative disease in which specific degeneration of the substantia nigra is the hallmark histopathological sign [1]. The substantia nigra contains non-pigmented and pigmented neurons, with the latter being more susceptible to degeneration in PD [2, 3]. The pigmented neuronal body contains a melanin-like granular pigment called neuromelanin (NM). NM gradually accumulates with increasing age but is decreased in PD cases compared to age-matched cases [4, 5]. NM-sensitive magnetic resonance imaging (NM-MRI) is a biomarker being developed to non-invasively identify patients in the prodromal stage of PD by attempting to detect the NM loss before the onset of clinical PD. However, quantification is made complex by the co-dependence of the MRI signal on NM and metal concentrations [6, 7]. Establishing a relationship between changing metal concentrations and NM concentrations, using an independent and quantitative NM assay, can progress the development of this biomarker. Quantifying NM concentration in the substantia nigra can also improve our currently limited knowledge about how different factors such as age, sex, and PD affect NM, and could provide important insights regarding NM’s role in the brain. However, NM is difficult to study due to its insolubility in most solvents except alkaline solutions such as sodium hydroxide. This makes quantification difficult since most assays require the analyte to be dissolved in an aqueous or non-polar solvent, and will not work at the high pH levels needed to solubilise NM [8, 9].

### Quantifying neuromelanin: Current methods

A common method to estimate NM content is using light microscopy, followed by stereology. Thin sections of the substantia nigra or locus coeruleus are cut, and pigmented neurons are counted using stereological methods [10, 11]. The section can be unstained or stained. However, increased contrast helps with counting, so it is common to use stains such as Cresyl violet, Fontana-Mason, or Haematoxylin & Eosin [12, 13]. This method estimates the number of pigmented neurons which can then be used to infer NM concentrations, so it is only semi-quantitative. Another disadvantage of stereology is that it is time inefficient, and differences in the counting methodology used can lead to differences in the quantification [5, 14]. Additionally, since neuronal NM content varies, pigmented neurons with little NM are given the same weighting as neurons with a lot of NM. Therefore, stereological counting cannot accurately represent NM content since it can only account for the absence or presence of the pigment [10, 11]. The stains can also increase the contrast of other components in the neurons, such as hemosiderin which appears similar to NM [15, 16]. Although relatively easy to do, the disadvantages and semi-quantitative nature of stereology does not make it a good method for NM quantification. Ideally, NM would be directly measured, and the process would be time efficient and unbiased. To the best of our knowledge, only one such methodology can be found in the literature [5]. Here substantia nigra was digested using Proteinase K, leaving behind NM. Then, the NM residue was washed, solubilised in sodium hydroxide, and the absorbance at 350 nm (A_350_) was measured using a spectrophotometer. This methodology was based on similar methods for quantifying melanin in skin cells [17]. The authors’ successful methodology showed that NM concentration in the substantia nigra increased with age in males and females. PD patients had lower NM concentrations than age-matched controls, and this finding agreed with previous histological assessments [5, 10].

### Optimising the digestion and spectrophotometric measurement of neuromelanin

A disadvantage of the relatively old methodology by Zecca and collaborators (2002) was that it required a minimum of 1 mL of the analyte since a cuvette spectrophotometer was used [5]. This limited sensitivity and the dynamic range of measurements. At least 10 mg of substantia nigra tissue was needed for measurements due to the relatively high analyte volume requirement. The scarcity of substantia nigra tissue and the destructive nature of the protocol meant that it may not always be possible to use this amount of tissue. Furthermore, the original methodology was limited to fresh-frozen brain tissue and did not work with formalin-fixed tissue. Since fresh-frozen brain tissue is scarcer and more difficult to store, being able to quantify NM in formalin-fixed tissue is greatly beneficial.

We optimised the technique to use smaller final volumes and less brain tissue. An acetone wash was also included to remove lipids, as leftover lipids affect the A_350_ measurements. We also included a tissue lysis solution to enhance the breakdown of the formalin-fixed tissue prior to protease digestion. The timings of steps were optimised throughout the protocol for efficiency. Our method enables easy upscaling of the number of tissue samples that can be analysed simultaneously for NM concentration; we successfully analysed up to 100 tissue samples at a time. A calibration curve is required to convert raw A_350_ measurements to NM concentrations. To create the calibration curve, the original protocol used NM extracted from fresh-frozen substantia nigra, but our protocol uses a robust process for synthesising NM [5, 18–20]. The synthetic NM is aged using high-heat to modify the structure to closer resemble endogenous NM [18]. Synthetic NM is a valid choice and has many advantages over SN NM (substantia nigra neuromelanin). Firstly, we avoid the digestion of tens of human SNs which renders the tissue unsuitable for further use and is thus wasteful. Secondly, we have found that the visible-ultraviolet absorbance spectra of SN NM and our synthetic NM are similar which makes the synthetic NM suitable for our purpose. Finally, the synthesis process is straightforward and utilises readily available precursors such as dopamine and L-cysteine.

The dependence of the final readout on enzymatic digestion is a possible limitation. Therefore, it is essential to completely digest the tissue, so care must be taken to break apart the formalin-fixed tissue. The digestion solution containing Proteinase K must also be made fresh. This methodology requires a spectrophotometer that can analyse less than 200 μL of analyte.

Modifying the original protocol from Zecca and colleagues (2002) allowed NM to be quantified in formalin-fixed samples, which was not previously possible. Absolute quantification of NM will help us better understand how NM changes with age, and in PD. Combined with metals analysis, this can help us better understand how both NM and metals influence the NM-MRI signal. This could be used to confirm if the decreased NM-MRI signal in PD patients can be wholly attributed to neuronal loss, thus further progressing the development of NM-MRI as an imaging biomarker for PD. In addition, while little is known about the function of NM, quantifying and comparing NM concentrations across various diseases can allow us to better understand its role in PD. The NM quantification method we described here is quantitative, efficient, and effective for measuring NM concentrations in formalin-fixed pigmented brain regions.

## Materials and methods

The protocol described in this article is published on protocols.io, dx.doi.org/10.17504/protocols.io.14egn29bpg5d/v1 [21]. It is also included for printing as S1 File with this article.

### Human brain tissue and ethics

The project was approved by the Human Anatomy Research committee (Approval # HAL00222022). The human brains used were sourced from the Department of Anatomy and Medical Imaging at the University of Auckland. These were part of the Human Body Bequest Program, where bodies are donated to be used for medical research and study, and is governed by New Zealand’s Human Tissue Act 2008. The process is carried out under the authority and guidance of the Inspector of Anatomy from the New Zealand Police. Written, informed consent to use the tissue for research was obtained from the individual(s) prior to their death and from the next of kin after death.

The Parkinson’s disease patient received an advanced Parkinson’s disease diagnosis and showed characteristic macroscopic reduction of myelin in the SN.

### Validating the synthetic neuromelanin

To validate our synthetic NM, the elemental composition, as % of carbon, hydrogen, nitrogen and sulphur (CHNS), of the synthetic NM was analysed using the Elementar vario EL cube. The dried, synthetic NM was ground in a fine powder using a glass rod. Approximately 5 mg of the sample was placed in a 4x4x11 mm tin boat (S22137418, Elementar) and rolled into a small ball. 5 mg of the reference standard, Sulfanilamide (S15.00–0062, Elementar), was also placed in the tin boats and rolled up. Analysis was as follows: 3x empty runs to purge gasses, 2x blanks with only rolled tin boats, 2x sulfanilamide standards, 7x NM samples, and 1x standard. Samples are burned in a combustion furnace, with oxygen, to yield N_x_O_y_ (reduced to N_2_), CO_2_, H_2_O, and SO_2_. Gasses are detected using the thermal conductivity detector. Blanks are subtracted from the samples, and % composition of CHNS in the synthetic NM is given.

To further validate the synthetic NM, we also measured and recorded the ultraviolet-visible absorption spectrum (200 nm– 700 nm) for both synthetic NM and SN NM.

### Converting the raw A350 measurement to NM concentration

For tissue NM quantification, the measured A_350_ of the solution must be converted into NM concentration using a standard curve. The process is as follows:

1. Average the A_350_ of the negative controls and subtract from all measurements.
2. Divide the A_350_ measurement by the calibration curve gradient to calculate the solution’s NM concentration.
3. Multiply by the final volume of the analysed solution (0.2 mL) to give the mass of NM in the solution.
4. Divide the NM mass by the starting mass of tissue to give the concentration of NM in the original tissue.

The data were normally distributed and passed the Shapiro-Wilk test for normality (p>0.05). Data were analysed in GraphPad Prism v9.0.0 using a one-way ANOVA with Tukey-HSD post hoc analysis; alpha was 0.05.

## Expected results

The parameters of our methodology were as follows: accuracy was 75.7% and interassay coefficient of variation was 6.75% (n = 5). Values were derived from tubes spiked with 3 μg synthetic NM that was processed identically and in parallel with the human SN samples. Accuracy was lower than expected since there would have been a greater loss of the NM due to the lack of brain tissue available for the NM to bind to. We chose not to spike the synthetic NM into homogenised brain tissue, such as the putamen, because there is evidence of NM in regions other than the SN and locus coeruleus [22].

### Extracting and quantifying neuromelanin: An overview

**Fig 1:**
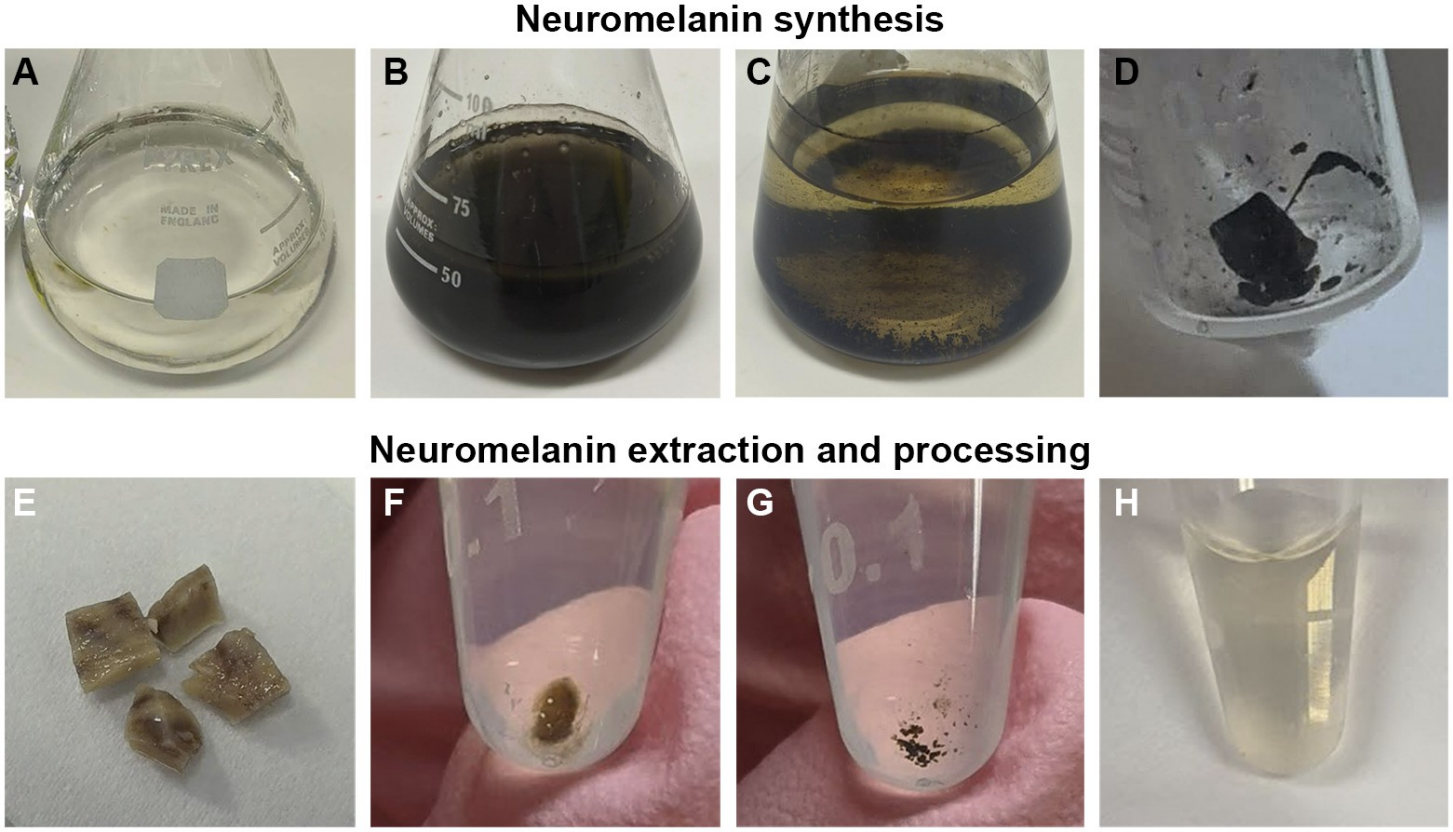
Various stages of neuromelanin synthesis, extraction, and processing. **(A)** Clear solution with dopamine, L-cysteine, and mushroom tyrosinase in phosphate buffer. **(B)** Synthesised neuromelanin in solution after 48 hours of incubation. **(C)** Synthesised neuromelanin in solution after 16 hours of high heat. **(D)** Dried and frozen synthetic neuromelanin granules. E-H shows the various stages of the quantification protocol. **(E)** dissected substantia nigra from a formalin-fixed human brain. **(F)** Pellet after overnight digestion with Proteinase K, and centrifugation. **(G)** Neuromelanin granules after washing with acetone, and centrifugation. **(H)** Solution with neuromelanin dissolved in sodium hydroxide.

NM inside the pigmented neurons must first be extracted and then dissolved in alkali, such as sodium hydroxide, for absolute quantification (Fig 1E–1H). The process starts with formalin-fixed human substantia nigra tissue, weighing between 2 and 20 mg (Fig 1E). After Proteinase K digestion (Fig 1F), an acetone wash is required to dissolve leftover lipids (Fig 1G). We found that lipids increased the A_350_ measurement of the solution, leading to artificially higher NM concentrations. Acetone was chosen because it is miscible with water which allows for easy removal from the tube, unlike other solvents such as hexane and chloroform. After solubilisation in sodium hydroxide (Fig 1H), the A_350_ measurement of the solution is measured using a microvolume spectrophotometer.

### Neuromelanin synthesis validation and calibration curve

The results from the elemental analysis of the % composition was as follows (mean ± SD): Carbon = 51.25± 0.49, Hydrogen = 4.13 ± 0.04, Nitrogen = 9.56 ± 0.09, and Sulphur = 7.01 ± 0.08. Our values are in line with % CHNS content of SN NM: Carbon = 58.80, Hydrogen = 7.16, Nitrogen = 6.51, and Sulphur = 2.56 [5]. The biggest difference was increased Sulphur in the synthetic NM.

**Fig 2:**
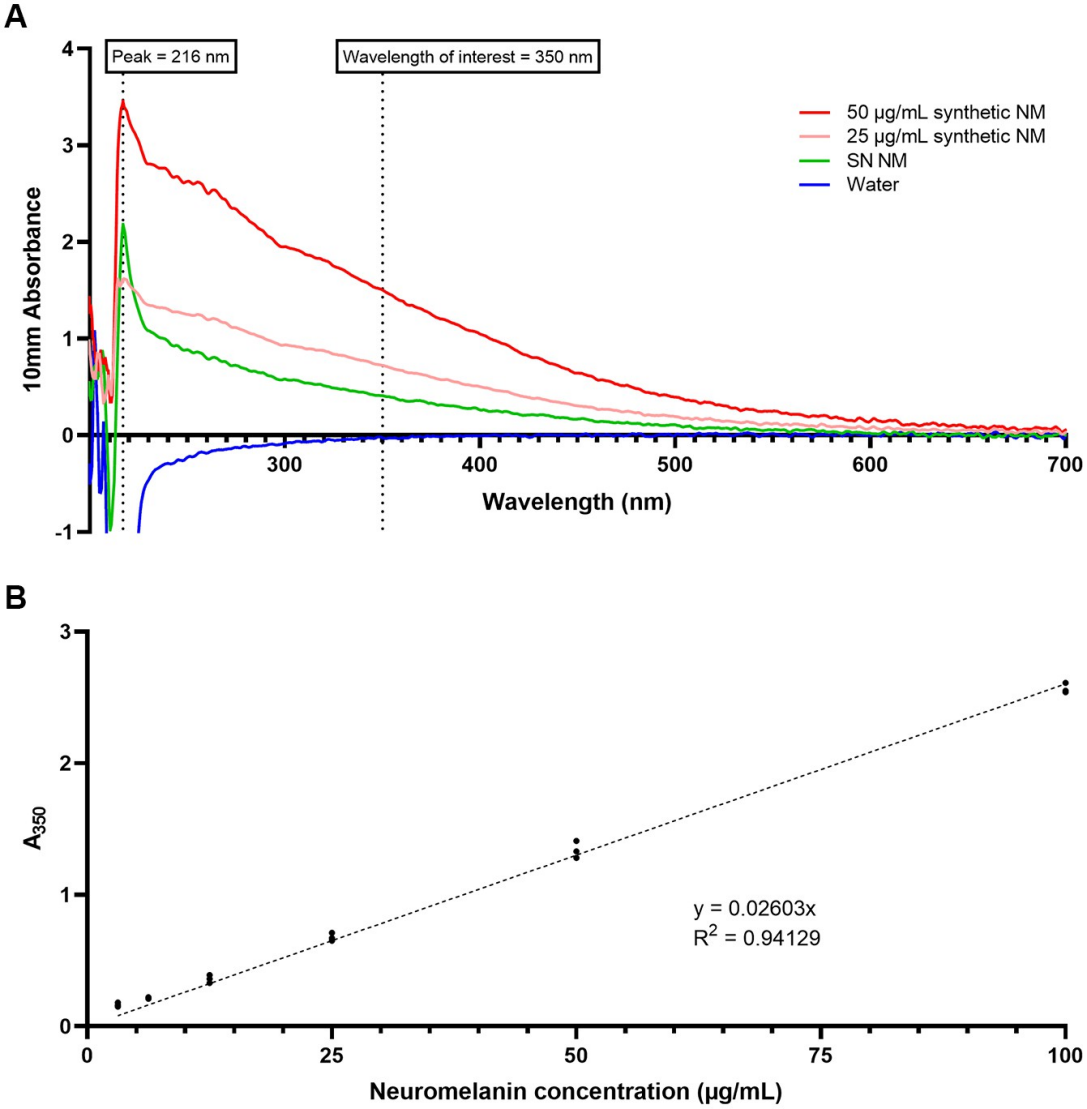
**(A) Ultraviolet visible spectra of synthetic and substantia nigra (SN) neuromelanin (NM)**. 2 M sodium hydroxide was used as the solvent and the blank, reference solution. Each spectrum is an average of n = 3 solutions. **(B) Calibration curve for neuromelanin quantification**. Different amounts of neuromelanin were dissolved in sodium hydroxide, and the absorbance at 350 nm (A_350_) was measured. Linear regression was performed and forced through y = 0 to give an equation. Data are shown as individual points, n = 3.

As per Fig 2A, the shape of the spectrum for synthetic and SN NM, in 2 M sodium hydroxide, are similar. In addition, the peak absorbance for both samples are identical, at 216 nm. The concentration of the SN NM was not known as it was not possible to weigh the small amount of extracted NM, but we expect the concentration to be 16 ug/mL, as back-calculated from the calibration curve. The calibration curve converts the raw absorbance values to NM concentrations, and its range was from 3.125 μg/mL to 100 μg/mL, which was appropriate for NM concentrations found in the formalin-fixed human substantia nigra. Linear regression was carried out, with the y-intercept forced at y = 0 since the A_350_ of a solution with no NM is expected to be 0 μg/mL (see Fig 2B). The R^2^ value was 0.941.

### Neuromelanin concentrations in the analysed brains

The substantia nigra from three formalin-fixed human brains were analysed using the protocol described in this paper; three samples were taken from each substantia nigra, from each side of the midbrain. The supporting information includes details about the donors (S1 Table). The raw A_350_ measurements and the initial masses of the analysed samples can also be found in the S2 Table.

**Fig 3:**
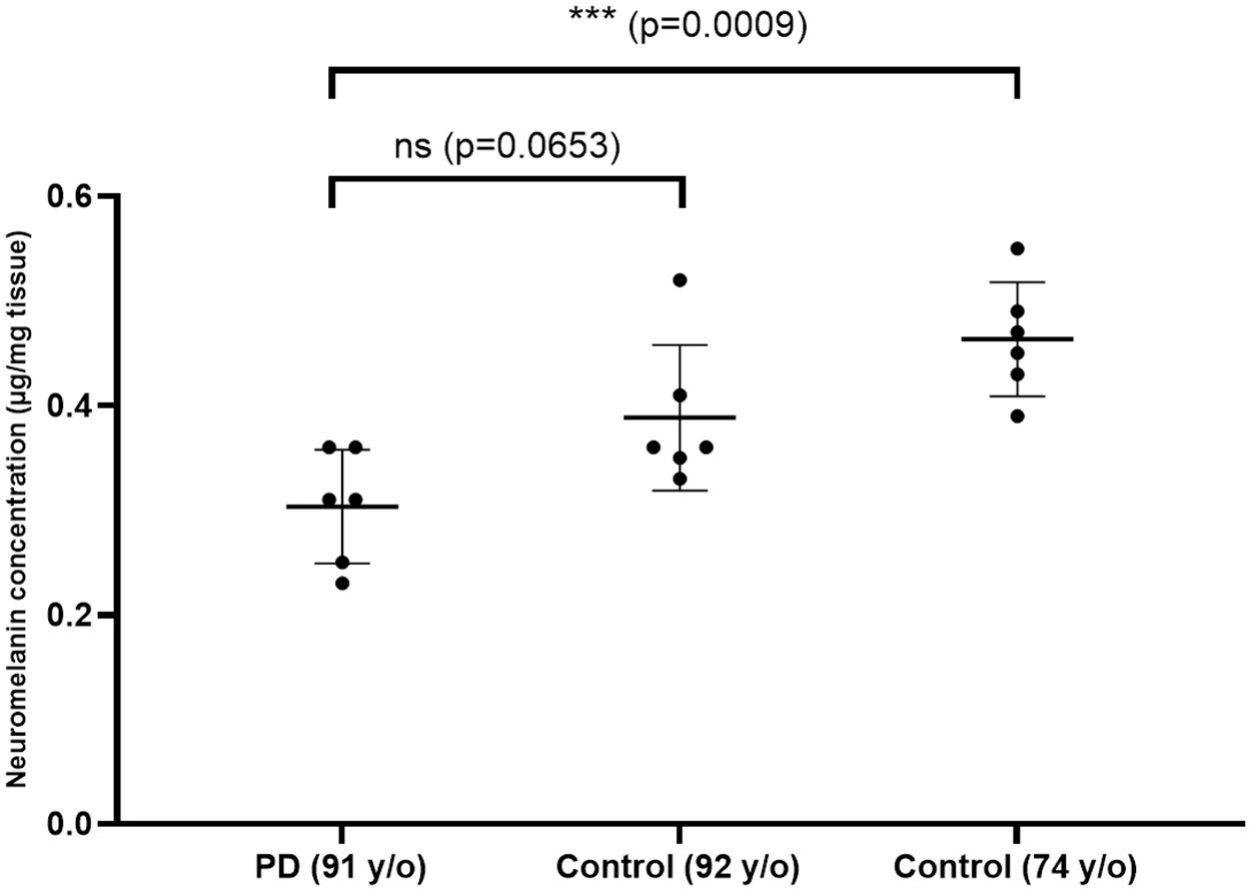
Neuromelanin concentration in the substantia nigra of three formalin-fixed brains. Three samples from the left and right hemispheres were combined and analysed (n = 6). Data are shown as mean ± standard deviation and were analysed using a one-way ANOVA with Tukey-HSD post hoc analysis. PD: Parkinson’s disease, y/o: years old.

The mean NM concentration (presented as μg/mg tissue ± standard deviation) in the PD brain (0.303 ± 0.054) was lower than in the 92-year-old control (0.388 ± 0.070), but this was not statistically significant (p = 0.0653). We suspect this may be due to other factors that influence NM concentrations such as race or comorbidities [23]. Conversely, since these brains came from very old subjects, age-related neurodegeneration may mask the PD-related SN degeneration. The mean NM concentration in the PD brain was significantly lower (p = 0.0009) than in the 74-year-old control (0.463 ± 0.055), and there was no significant difference between the two control brains (Fig 3). The measured values generated using the protocol are a straightforward way to compare NM concentration between different formalin-fixed brains which can give valuable insights about NM changes in PD or ageing.

The locus coeruleus can be identically analysed using this technique. Care must be taken to dissect the intended region of interest due to the small size and thin shape of the locus coeruleus.

## Supporting information

S1 File Step-by-step protocol, also available on protocols.io. (PDF)

S1 Table Details about the human donor brains. Some information may be omitted due to privacy. TSD = time since death/fixation (years).
(DOCX)

S2 Table Raw absorbance values of samples processed using the NM quantification protocol. PD: Parkinson’s disease. A_350_: absorbance at 350 nm.
(DOCX)
