# Supplementary material for: Absolute quantification of neuromelanin in formalin-fixed human brains using absorbance spectrophotometry
Source: PLoS One. 2023 Jul 10;18(7):e0288327. doi: 10.1371/journal.pone.0288327 (PMC10332574; doi:10.1371/journal.pone.0288327)
Supplement: S1 File — (PDF) [file pone.0288327.s001.pdf]

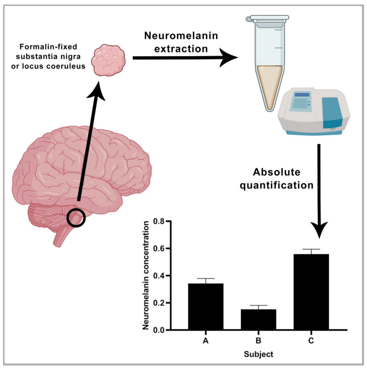

CR4EV8TE

Protocol Info: Dipshay Avi Chand, Miriam Scadeng, Birger Victor Dieriks . Neuromelanin quantification in formalin-fixed substantia nigra and locus coeruleus. **protocols.io** https://protocols.io/view/neuromelanin-quantification-in-formalin-fixed-subs-cr4ev8teVersion created by Dipshay A Chand

Created: Mar 29, 2023

Last Modified: May 02, 2023

PROTOCOL integer ID: 79718

Keywords: neuromelanin, parkinson's disease, absolute quantification, substantia nigra

Neuromelanin quantification in formalin-fixed substantia nigra and locus coeruleus V.(cr4ev8te)

Dipshay Avi Chand[1,2], Miriam Scadeng[1,2], Birger Victor Dieriks[1,2]

[1]Department of Anatomy and Medical Imaging, University of Auckland, Auckland, New Zealand;
[2]Centre for Brain Research, University of Auckland, Auckland, New Zealand

D Dipshay A Chand

ABSTRACT

There are limited methods for absolute quantification of neuromelanin in brain regions such as the substantia nigra and locus coeruleus. While stereology is typically used to quantify neuromelanin in these regions, it is semi-quantitative.

A protocol for absolute quantification of neuromelanin using spectrophotometry was adapted for the modern laboratory. The process was improved so neuromelanin could be reliably quantified in formalin-fixed samples. A methodology to synthesise neuromelanin is also described, which prevents the wastage of substantia nigra neuromelanin since it is required for the calibration curve. The methodology described here is unbiased, high-throughput, and can measure neuromelanin concentration reliably.

MATERIALS

Laboratory equipment
- 50 mL polypropylene conical centrifuge tube (14-432-22, Corning)
- Mini plastic pestle, for homogenisation (F19923-0001, SP Bel-Art)
- 1.5 mL microcentrifuge tubes, with cap (1210-10, SSI Bio)
- Vortex mixer (K-550-GE, Vortex Genie)
- Ultrasonic water bath (FXP 10DH, Unisonics)
- Absorbance spectrophotometer (NanoDrop One, Thermo Fisher Scientific)

Biological material
- Formalin-fixed human substantia nigra (or locus coeruleus), dissected into 2-10 mg pieces

Chemicals and reagents
- Sodium phosphate monobasic (≥99.0% powder, S0751, Sigma-Aldrich)
- Sodium phosphate dibasic (≥99.0% powder, S0876, Sigma-Aldrich)
- Dopamine hydrochloride (≥98% powder, H8502, Sigma-Aldrich)
- L-cysteine (97% powder, 168149, Sigma-Aldrich)

- 1 M hydrochloric acid (1 M solution, J-4320-17, Fisher Chemical)
- Mushroom tyrosinase lyophilised powder (T3824, Sigma-Aldrich)
- Acetic acid (glacial, 10401, ECP Labchem)
- Sodium dodecyl sulfate (≥99.0% pellets, 75746, Sigma-Aldrich)
- Tris base (≥99.9% powder, 15504020, Invitrogen)
- Sodium chloride (≥99.7% crystals, BSPSL944, LabServ)
- Acetone (≥99.8% solution, 2440-16, ECP Labchem)
- Proteinase K lyophilised powder (≥90% crystals, P1264, AG Scientific)
- Sodium hydroxide (≥98% pellets, S5881, Sigma-Aldrich)
- Calcium chloride dihydrate (≥99.0% crystals, C8106, Sigma-Aldrich)
- Ultrapure water (Milli-Q, Merck-Millipore)
- Nitrogen gas (BOC Healthcare)

Buffers and reagent stocks
- Phosphate buffer (50 mM sodium phosphate monobasic + 50 mM sodium phosphate dibasic, in water, pH 7.4)
- Dopamine stock (79 mM (15 mg/mL) dopamine hydrochloride, in water)
- L-cysteine stock (0.41 M (50 mg/mL) L-cysteine, in 1 M hydrochloric acid)
- Mushroom tyrosinase stock (8503 units/mL, in water)
- 33% acetic acid (diluted from glacial acetic acid, in water)
- Tris buffer (17 mM (5 mg/mL) sodium dodecyl sulfate + 50 mM Tris base, in water, pH 7.4)
- Sodium chloride solution (0.17 M (10 mg/mL) sodium chloride, in water)
- Tissue lysis solution (0.17 M sodium dodecyl sulfate + 400 mM Tris base, in water, pH 9.0)
- Sodium hydroxide solution (4 M sodium hydroxide, in water)

* Prepare Proteinase K stock solution from the lyophilised powder as per manufacturer's instructions (we created a 10 mg/mL Proteinase K stock solution in 50 mM Tris base + 1 mM calcium chloride dihydrate, in water, pH 8.0)

BEFORE START INSTRUCTIONS

This protocol will use an absorbance spectrophotometer that can measure volumes less than 200 µL.

Prepare all required buffers and solutions before starting (see materials section).

If you already have dried neuromelanin (synthetic or endogenous), skip to step 13.

Neuromelanin synthesis

1

Note

The reagent volumes listed here yields 10 mg of neuromelanin.

The room temperature in the lab was 22°C.

Add 50 mL phosphate buffer, 630 µL dopamine stock solution, 61 µL L-cysteine stock solution, and 250 µL mushroom tyrosinase stock to an Erlenmeyer flask

2 Wrap the flask with aluminium foil and make small holes on top; this protects the reaction from light but allows exposure to air. Put the flask in an incubator for 48 hours, at 37°C, with constant shaking (120 rpm).

Expected result

The solution should appear dark at the end of the incubation.

3 Stop the reaction by decreasing the solution pH to between 3 and 4, using 33% acetic acid.

4 Keep the solution wrapped in foil, as before. Incubate for 16 hours at 95°C, in an oven.

Expected result

The solution should remain dark, but the pigment should appear more granular and settled at the bottom.

5 Transfer the solution into 50 mL polypropylene tubes. Centrifuge the solution for 15 minutes at 3800 x g. Pour off the supernatant and transfer the slurry at the bottom into a 2 mL microcentrifuge tube.

Note

The room temperature in the laboratory space was 22°C.

6 Add 1.5 mL Tris buffer and incubate for 2 hours at 37°C.

7 Centrifuge the tube for 2 minutes at 9000 x g. Pour off supernatant.

8 Add 1.5 mL sodium chloride solution and wash the pellet. Centrifuge the tube for 2 minutes at 9000 x g. Pour off supernatant.

9 Add 1.5 mL ultrapure water and wash the pellet. Centrifuge the tube for 2 minutes at 9000 x g. Pour off supernatant.

Repeat this step once more.

10 Add 1.5 mL acetone and wash the pellet. Centrifuge the tube for 2 minutes at 9000 x g. Pour off supernatant.

Expected result

A small amount of acetone will be left behind, with the clean neuromelanin dispersed throughout.

11 Dry the neuromelanin under flowing nitrogen gas for about 24 hours.

Expected result

The acetone should be fully evaporated, and the neuromelanin should be completely dry.

The expected yield is ~80% of the starting mass of materials.

12 Store the synthetic neuromelanin at -20°C in a closed plastic tube that is wrapped with aluminium foil to prevent light exposure.

Create a neuromelanin stock solution

13

Note

We recommend making a 0.5 mg/mL stock solution of neuromelanin to make the process of creating the calibration curve easier. This stock can also be used for positive controls in the quantification process.

Add the frozen, dried neuromelanin to ultrapure water, adjust to pH 9.0. Sonicate in an ultrasonic ice water bath for 20 minutes. Store this solution at 4°C until use.

Quantify neuromelanin in a brain region

14

Note

These instructions are for analysing a single sample of brain tissue (2-20 mg). However, many samples can be easily analysed in parallel; prepare the necessary volumes of buffers and reagents as needed (see materials section).

Weigh 2-20 mg of fixed brain tissue (substantia nigra or locus coeruleus) using a high-accuracy scale. Homogenise the tissue in a 1.5 mL tube, using a mini plastic pestle, until a relatively fine homogenate is formed.

Note

Negative controls can be included, which are processed the same as the samples, except no brain tissue or synthetic neuromelanin is added at the start.

Positive controls can also be included, which are processed the same as the samples, except a known amount of neuromelanin is spiked at the start.

15 Add 1000 µL tissue lysis buffer, cap tubes, and incubate in a dry heating block for 90 minutes at 90°C. Shake tube halfway through incubation.

Centrifuge the tube for 10 minutes at 15,000 x g.

16 Remove 900 µL of the supernatant using a micropipette, leaving behind 100 µL in the tube. Add 900 µL phosphate buffer.

Centrifuge the tube for 10 minutes at 15,000 x g.

16.1 Create the Proteinase K digestion solution by adding Proteinase K to Tris buffer to achieve a concentration of 0.25 mg/mL Proteinase K. Keep solution on ice.

17 Remove 900 µL of the supernatant. Add 900 µL Proteinase K digestion solution. Incubate the tube overnight (12-14 hours) at 50°C, with constant shaking (130 rpm).

Note

Laying the tube along its length is recommended to allow adequate dispersion and digestion of the tissue.

17.1 Centrifuge the tube for 10 minutes at 12,000 x g.

18 Remove 900 µL of the supernatant, leaving behind 100 µL in the tube. Add 900 µL sodium chloride solution.

Centrifuge the tube for 10 minutes at 12,000 x g.

Expected result

There should be a dark pellet at the bottom of the tube. This is the neuromelanin that was in the original brain sample. The surrounding tissue has been digested by Proteinase K.

19 Remove 950 µL of the supernatant, leaving behind 50 µL. Add 450 µL acetone.

Wait for 5 minutes.
Vortex the tube for ~5 seconds.
Place in a room-temperature ultrasonic water bath for 3 minutes.

Centrifuge the tube for 10 minutes at 12,000 x g.

20 Remove 450 µL of the supernatant. Add 950 µL ultrapure water.

Centrifuge the tube for 10 minutes at 12,000 x g.

21 Remove 900 µL of the supernatant, leaving behind 100 µL. Add 100 µL 4 M sodium hydroxide. The final volume should be 200 µL.

Sonicate the tube in a room-temperature ultrasonic water bath for 5 minutes.

22 Incubate for 1 hour at 80°C. Shake halfway through the incubation period.

Expected result

At the end of the incubation, no neuromelanin granules should be left at the bottom of the tube. If there is, increase the incubation period until all the neuromelanin has been dissolved.

23 Centrifuge the tube for 10 minutes at 15,000 x g.

Measure the absorbance at 350 nm using an absorbance spectrophotometer; we used the NanoDrop One (Thermo Fisher Scientific). Use 2 M sodium hydroxide as the blank solution.

Neuromelanin calibration curve

24

Note

The standard curve that is suggested here is from a concentration range of 3.125 µg/mL to 100 µg/mL. It uses the synthetic neuromelanin stock solution created earlier. The neuromelanin concentrations found in the pigmented brain regions fall within the concentration range.

In triplicate, add 160 µL of the 0.5 mg/mL neuromelanin stock solution, 40 µL ultrapure water,

and 200 µL 4 M sodium hydroxide into a 1.5 mL tube. This is a 200 µg/mL solution.

Incubate in a dry heating block for 1 hour at 80°C. Centrifuge for 5 minutes at 15,000 x g.

25 Starting with the 200 µg/mL solution, use two-fold serial dilutions to create solutions that are 100, 50, 25, 12.5, 6.25, and 3.125 µg/mL. Use 2 M sodium hydroxide as the diluent.

26 Measure the absorbance at 350 nm using an absorbance spectrophotometer. Use 2 M sodium hydroxide as the blank solution.

Expected result

This data can be used to generate a calibration curve which can then be used to relate sample absorbance at 350 nm to known NM concentration.
