# Supplementary material for: Absolute quantification of neuromelanin in formalin-fixed human brains using absorbance spectrophotometry
Source: PLoS One. 2023 Jul 10;18(7):e0288327. doi: 10.1371/journal.pone.0288327 (PMC10332574; doi:10.1371/journal.pone.0288327)
Supplement: S1 Table — Some information may be omitted due to privacy. TSD = time since death/fixation (years). (DOCX) [file pone.0288327.s002.docx]

**S1 Table. Details about the human donor brains.** Some information may be omitted due to privacy. TSD = time since death/fixation (years).

| **Label** | **Age** | **Sex** | **Cause of death** | **TSD** |
| --- | --- | --- | --- | --- |
| PD | 91 | Male | Aspiration pneumonia | 8 |
| Control (92 y/o) | 92 | Male | Pneumonia | 3 |
| Control (74 y/o) | 74 | Male | Respiratory infection | 3 |
