# Supplementary material for: Absolute quantification of neuromelanin in formalin-fixed human brains using absorbance spectrophotometry
Source: PLoS One. 2023 Jul 10;18(7):e0288327. doi: 10.1371/journal.pone.0288327 (PMC10332574; doi:10.1371/journal.pone.0288327)
Supplement: S2 Table — PD: Parkinson’s disease. A350: absorbance at 350 nm. (DOCX) [file pone.0288327.s003.docx]

**S2 Table.** **Raw absorbance values of samples processed using the NM quantification protocol.** PD: Parkinson’s disease. A_350_: absorbance at 350 nm.

| **Brain** | **Hemisphere** | **A_350_** | **Tissue mass (mg)** |
| --- | --- | --- | --- |
| PD (91 y/o) | Left | 0.21 | 8.9 |
|  |  | 0.31 | 10.5 |
|  |  | 0.25 | 8.6 |
|  | Right | 0.22 | 6.5 |
|  |  | 0.61 | 18.2 |
|  |  | 0.35 | 15.8 |
| Control (92 y/o) | Left | 0.29 | 6.0 |
|  |  | 0.31 | 9.1 |
|  |  | 0.15 | 4.4 |
|  | Right | 0.19 | 5.7 |
|  |  | 0.44 | 14.2 |
|  |  | 0.33 | 8.5 |
| Control (74 y/o) | Left | 0.20 | 4.4 |
|  |  | 0.32 | 7.1 |
|  |  | 0.60 | 11.7 |
|  | Right | 0.43 | 10.2 |
|  |  | 0.69 | 17.3 |
|  |  | 0.48 | 13.3 |
